# Decoding anxiety: A scoping review of observable cues

**DOI:** 10.1177/20552076241297006

**Published:** 2024-11-26

**Authors:** Urška Smrke, Izidor Mlakar, Ana Rehberger, Leon Žužek, Nejc Plohl

**Affiliations:** 1 Faculty of Electrical Engineering and Computer Science, University of Maribor, Maribor, Slovenia; 2 Department of Psychology, Faculty of Arts, University of Maribor, Ljubljana, Slovenia; 3 Center for Cognitive Science, Faculty of Education, University of Ljubljana, Ljubljana, Slovenia; 4 Department of Psychology, Faculty of Arts, University of Maribor, Maribor, Slovenia

**Keywords:** Anxiety, observable cues, digital biomarkers, scoping review, physiological cues, behavioural cues

## Abstract

**Background:**

While anxiety disorders are one of the most prevalent mental diseases, they are often overlooked due to shortcomings of the existing diagnostic procedures, which predominantly rely on self-reporting. Due to recent technological advances, this source of information could be complemented by the so-called observable cues – indicators that are displayed spontaneously through individuals’ physiological responses or behaviour and can be detected by modern devices. However, while there are several individual studies on such cues, this research area lacks a synthesis. In line with this, our scoping review aimed to identify observable cues that offer meaningful insight into individuals’ anxiety and to determine how these cues can be measured.

**Methods:**

We followed the PRISMA guidelines for scoping reviews. The search string containing terms related to anxiety and observable cues was entered into four databases (Web of Science, MEDLINE, ERIC, IEEE). While the search – limited to English peer-reviewed records published from 2012 onwards – initially yielded 2311 records, only 33 articles fit our selection criteria and were included in the final synthesis.

**Results:**

The scoping review unravelled various categories of observable cues of anxiety, specifically those related to facial expressions, speech and language, breathing, skin, heart, cognitive control, sleep, activity and motion, location data and smartphone use. Moreover, we identified various approaches for measuring these cues, including wearable devices, and analysing smartphone usage and social media activity.

**Conclusions:**

Our scoping review points to several physiological and behavioural cues associated with anxiety and highlights how these can be measured. These novel insights may be helpful for healthcare practitioners and fuel future research and technology development. However, as many cues were investigated only in a single study, more evidence is needed to generalise these findings and implement them into practice with greater confidence.

## Introduction

In 2019, 301 million people around the world were living with an anxiety disorder, which is characterised by excessive or persistent feelings of anxiety.^1,2^ More specifically, it is marked by anticipation of future threats and accompanied by symptoms of worry, avoidance, restlessness and muscle tension.^2,3^ It can significantly negatively impact the quality of individuals’ lives.^4^ According to the study of epidemiological data,^5^ the number of people affected by anxiety has increased by 55% from 1990 to 2019. Additionally, a significant increase in anxiety has happened due to the COVID-19 outbreak in 2020^6^ and the onset of the Russian–Ukrainian war at the beginning of 2022.^7^ As such, it has reached epic proportions across the globe.

With such widespread anxiety and other mental health issues, their early recognition is of utmost importance. However, at the primary care level, several factors possibly contribute to low rates of anxiety disorder identification. A meta-analysis^8^ has shown that general practitioners’ sensitivity to recognising anxiety in an individual is relatively low (44.5%). While it can be argued that the education and skills for recognising common mental health problems are improving in non-psychiatric physicians, based on publications describing recent efforts in this direction (e.g., refs.^9–11^), several studies still report opportunities for improvement. For example, Canadian physicians reported that they would benefit from more education and additional support on the topic,^12^ Australian general practitioners reported self-identified gaps in their knowledge related to recognition of mental health problems,^13^ and in Greece, more than half of general practitioners in the sample identified their education on the topic as insufficient.^10^ A lack of education can lead to the underestimation of the possibility of a mental health disorder.^14^

Another set of barriers stems from individuals with potential mental health problems. Since anxiety is related to more frequent reports of somatic symptoms,^15^ patients often turn their focus to the physical component and fail to acknowledge that their symptoms may be of psychological origin.^16,17^ Moreover, individuals may see the symptoms as part of their personality and not consider that they are out of the ordinary.^17^ Sometimes, patients do not recognise the key symptom of anxiety (i.e., excessive, persistent worry) and do not know how to articulate it during psychiatric interviews.^18^ Identifying the problem is especially challenging for those struggling with social anxiety, because people who experience this type of anxiety often avoid social contact and thus do not seek professional help, which mostly requires human contact.^14,19^ Similar barriers have been reported in a more recent systematic review on barriers related to seeking and accessing professional help for mental health problems among children and adolescents.^20^ In particular, the review identified limited health knowledge, negative perceptions of help-seeking, social factors (e.g., social stigma, embarrassment), perceived therapeutic relationship with professionals and systemic structural barriers (e.g., financial costs) as the main reasons for not seeking professional help. All these barriers may, in the next step, contribute to mental health problems remaining undiagnosed.^14,19^

Further issues in recognition of anxiety disorders may arise because screening of anxiety disorders usually relies on self-report questionnaires, which ask individuals to answer questions regarding symptoms most typically related to anxiety (i.e., uncontrollable worry or feeling nervous).^21^ Even though screening for anxiety symptoms through questionnaires is the most conventional approach, it also has some disadvantages. They may be time-consuming and generate false negatives.^22,23^ Moreover, questionnaires, especially those based on rating scales, may lead to response biases (e.g., social desirability and recall bias), significantly undermining the measurement's validity and thus magnifying the issue of anxiety underdiagnosis.^24–26^ Questionnaires are also administered only occasionally, possibly missing important changes between two consecutive measurements.^27^

In contrast to relying on self-report data to detect symptoms of anxiety, a growing field of research has recently turned its focus to observable cues, such as facial expressions and language characteristics, and relating them to individuals’ psychological characteristics.^28^ Due to the emergence of devices able to detect such cues and (explainable) artificial intelligence (AI) algorithms able to make predictions on mental health disorders, this approach now holds great promise for more timely and accurate screening for anxiety.^29^ Such advances present an important opportunity for more effective and accurate recognition of anxiety,^30^ potentially leading to earlier diagnosis and improving the lives of those affected.^31^ At the same time, they may contribute to managing the burden of healthcare professionals that could use such technology during patients’ visits or recommend home monitoring for their at-risk patients.

Observable cues are indicators that a person displays spontaneously and can be observed in their behaviour (e.g., specific language patterns, speech nuances, facial expressions) and are, in the case of anxiety, characteristic of people experiencing anxiety. As such, they are less likely to be impacted by cognitive and other biases.^28,30^ As they can be observed outwardly and do not rely (solely) on a person reporting on their internal state, they can be detected by a device independent of human judgment, making them useful in the process of screening for anxiety. Previous research has, for example, focused on mobile applications,^32–34^ wearable devices,^35,36^ smartphone location data,^37,38^ smartphone usage data,^39,40^ physiological data,^41–43^ and social media data,^44^ to detect such observable cues.

This review focuses on identifying observable cues that can be detected via widespread devices, such as wearable devices and smartphones. Such devices possess various sensing and computation capabilities, which can be used for detecting anxiety^38^ in natural environments (e.g., at home). In addition to that, they can be used to pick up continuous observable cues of anxiety in real time, as opposed to self-report questionnaires, which often demand reporting mental health issues retrospectively. As pointed out in a review,^45^ reporting health symptoms in retrospect could be influenced by memory errors. Thus, using technology to monitor anxiety in real time could significantly improve its recognition.

The aim of this study is to provide a scoping review of different types of observable anxiety cues explored in existing literature and methods of their measurement. Due to their objectivity, possibility of continuous data measurement and availability in various contexts (e.g., home), we believe such methods may potentially facilitate early recognition of anxiety disorders and their prevention. We also argue that such measurements could complement self-report questionnaires for anxiety screening and inform clinicians’ decisions in tertiary healthcare on the anxiety disorder diagnosis. To achieve our study aim, we formulated two research questions: (RQ1) *What are meaningful observable cues that can offer a valid insight into individuals’ anxiety?* and (RQ2) *How are those cues measured?* By providing answers to RQ1 and RQ2, this review may highlight further directions for research and the design of new methodologies and tools to facilitate a more informed and objective diagnosis of anxiety disorder.

## Methods

### Overview

The present scoping review was conducted in line with the framework proposed by Arksey and O’Malley,^46^ which consists of five stages, namely: identifying the research questions, identifying relevant studies, study selection, charting the data, and, lastly, collating, summarising and reporting results. Moreover, the results of our review are reported according to the PRISMA-ScR (Preferred Reporting Items for Systematic Reviews and Meta-Analyses Extension for Scoping Reviews) guidelines for scoping reviews.^47^

### Identifying the research questions

In the first step, we developed two research questions that guided our review set within the context of an EU-funded project that aims to develop a gamification-based platform capable of assessing and reducing individuals’ anxiety. Our primary research question was: (RQ1) *What are meaningful observable cues that can offer a valid insight into individuals’ anxiety?* Moreover, we set a secondary research question oriented towards the measurement of observable cues: (RQ2) *How are those cues measured?*

### Identifying relevant studies

We used four databases, specifically Web of Science, MEDLINE, ERIC and IEEE, to identify the relevant papers. These databases were chosen as they include a large number of articles, cover a wide range of disciplines, and are commonly used in other literature reviews tackling research questions similar to ours. After a preliminary search, which helped us adjust the strategy for optimal results, we conducted the main search on August 1st, 2023. Our search string combined terms related to anxiety (anxiety OR anxious OR phobia OR “panic disorder”) and various terms related to (potential) observable cues of anxiety (“digital biomarker*” OR “electronic biomarker*” OR “digital phenotyp*” OR “digital footprint*” OR “digital measure*” OR “human-smartphone interaction data” OR “smartphone data” OR sensor OR sensors OR “observable cue*” OR “physiological marker*” OR “behavioral cue*” OR “behavioral data” OR “physiological data”). These terms were both general (to identify a wide range of observable cues) and specific (to investigate observable cues previously explored in similar contexts). Articles were included in the initial search if they were peer-reviewed, available in English, and published in the last 10 years (i.e., in January 2012 or later). These inclusion criteria were chosen to ensure high-enough quality of articles and due to the past decade seeing significant advances in research methods, including more sophisticated sensors able of detecting and validly measuring the observable cues. To identify potential additional articles that were not found via primary databases, simplified combinations of search terms were also run in Google Scholar. Specifically, we searched for anxiety, “digital biomarkers” as well as anxiety, “observable cues”.

### Study selection

All citations identified in the electronic databases were exported to Microsoft Excel spreadsheets. We then performed the study selection procedure in two review stages. In the first step, two authors (AR and LŽ) individually screened the titles and abstracts of each paper, while two other authors (US and NP) performed an additional quality check (i.e., independent screening of randomly selected papers) and found no errors. In the second step, authors (US, AR, LŽ, IM and NP) independently reviewed full articles. Records were excluded (i.e., classified as not relevant) in the full-text review stage if they met the following criteria: (a) study not original and empirical (i.e., reviews of previously published research and studies without human participants were excluded), (b) participants with disorders that affect behaviour and display of emotions (e.g., autism, stroke, mutism^1^), (c) comorbidity reported (e.g., study explicitly reported that anxious participants also suffered from other disorders, such as depression), (d) associations between observable cues and anxiety not explored (e.g., focus on other phenomena, such as stress, or no results pertaining to the association between features and anxiety), (e) features cannot be observed objectively (e.g., only self-report), (f) special equipment needed to measure features (e.g., EEG, fMRI, salivary analyses) and (g) no data regarding individual features (i.e., only evaluation of composite algorithms available). The methodological quality of studies was not treated as a reason for exclusion. Moreover, it is worth noting that research articles that included multiple studies, recruited different subsamples, or used various devices to measure the observable cues were not excluded as long as they involved at least some data relevant for the present review.

**Figure 1.**
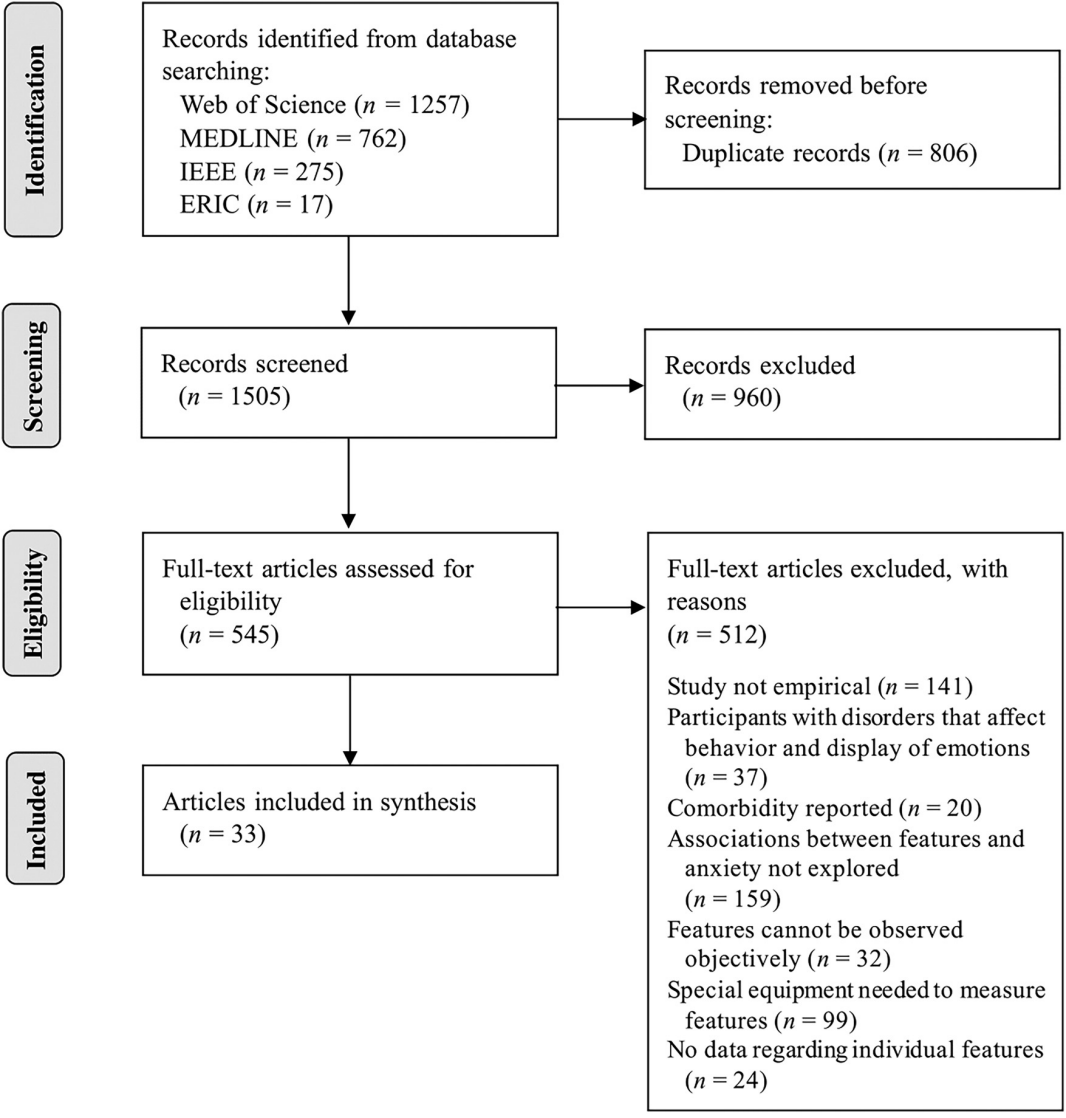
PRISMA flowchart depicting the study selection process.

As shown in Figure 1, our search led to 2311 English language peer-reviewed articles published in the last 10 years (Web of Science: 1257 hits, MEDLINE: 762 hits, IEEE: 275 hits, ERIC: 17 hits), whereas the additional search in Google Scholar did not result in any previously unidentified records. The four chosen databases overlapped significantly, which led to the removal of 806 records. After the first review stage, 545 articles (36.2% of identified unique records) still met our criteria. In contrast, only 33 articles (2.2% of identified unique records) fit our inclusion criteria after the second review stage and were hence included in the final synthesis.

### Charting the data

After completing both review stages, four authors (US, AR, LŽ and NP) independently extracted data from relevant papers using the same Microsoft Excel spreadsheet. The latter contained several headings that corresponded with our research questions, namely: authors, year, country, type of study (e.g., experimental, observational), sample size, sample description, information regarding the comparison group, type of anxiety, measure of anxiety, information regarding the diagnosis, observed cue of anxiety, description of key results, effect size (if available) and method of observation. Each observed anxiety cue was entered into a separate line in the spreadsheet.

### Collating, summarising and reporting results

The gathered data were analysed by two authors (US and AR) who categorised individual closely related observable cues into higher-order categories. Several iterations were performed to reduce the number of categories sufficiently. The process of collating, summarising and reporting results was reviewed by two authors (IM and NP).

## Results

In the present study, we reviewed 33 studies on observable cues of anxiety. Despite searching for articles published from 2012 onwards, most of the studies were published in the last 5 years (*n *= 28, 84.8%; 26, 28, 53, 55, 57–59, 61–65, 67–71, 73, 75–84). The majority of studies were conducted in North America (*n *= 10, 30.3%; 53, 57, 60, 64, 66, 72, 75, 78, 80, 84) or Europe (*n *= 9, 27.3%; 26, 28, 55, 63, 65, 67, 69, 71, 74), followed by those conducted in Asia (*n *= 8, 24.2%; 59, 61–62, 68, 70, 73, 76–77) and Australia (*n *= 1, 3.0%; 82). The remaining studies used international samples (*n *= 2, 6.1%; 56, 81) or did not contain information on geographical location (*n *= 3, 9.1%; 58, 79, 83).

Sample sizes of the reviewed studies ranged from seven to 1039 participants, with an average of 172.4 participants (*SD *= 238.2). Age-wise, most of the studies were conducted on adult participants (*n *= 19, 57.6%; 28, 53, 55–61, 65–66, 69, 72, 75–77, 81, 83–84), followed by students (*n *= 10, 30.3%; among which one study (3.0%) included only male students; 62–64, 67, 70, 73–74, 78–80), young adults (*n *= 2, 6.1%; 26, 71) and adolescents (*n *= 2, 6.1%; 68, 82). Most of the samples were drawn from the general population without explicitly including participants with anxiety (i.e., general adult population: *n *= 25, 72.7%; office employees: *n *= 1, 3.0%; health care professionals; *n *= 1, 3.0%; 28, 55–59, 61–68, 70–73, 75–78, 80–84). Four (12.1%; 26, 60, 74, 79) studies employed samples that reported having a diagnosis or symptoms of anxiety in the recruitment phase (i.e., whole sample or part of the sample), one study (3.0%; 53) employed a sample of adult participants with and without stuttering, and one study (3.0%; 69) adult participants who were suspected of having lung cancer.

Only one (3.0%; 60) reviewed study included individuals who were clinically diagnosed with some type of anxiety (i.e., social anxiety disorder by DSM-IV criteria), while another study (3.0%; 73) included participants who had self-declared social phobia. All other studies assessed anxiety via previously validated self-report questionnaires, most often State-Trait Anxiety Inventory (STAI^48^; *n *= 7, 21.2%; 59, 62–63, 66–67, 69, 71), Generalised Anxiety Scale (GAD^49^; *n *= 5, 15.2%; 57–58, 75, 79, 81), Liebowitz Social Anxiety Scale (LSAS^50^; *n *= 5, 15.2%; 57, 74, 76, 83–84) and Social Interaction Anxiety Scale (SIAS^51^; *n *= 5, 15.2%; 53, 64, 72, 78, 80). Other questionnaires were employed in three studies or less. Most studies (*n *= 26, 78.8%; 26, 28, 53, 56, 58–59, 61–63, 65–68, 70–80, 83–84) reported no comorbidities in their samples or the information regarding comorbidities was not available in the paper. Regarding comparison groups in the reviewed studies, most of them (*n *= 26, 78.8%; 26, 28, 55, 58–75, 77–80, 84) reported no comparison groups. In contrast, four studies (12.1%; 76, 81–83) categorised and compared participants based on anxiety scores obtained with self-report questionnaires, one (3.0%; 57) categorised them according to results of screening for mental illness, one (3.0%; 56) study induced anxiety and then differentiated between high and low anxiety conditions, and in one study (3.0%; 53), adults without stuttering (as a condition highly correlated with social anxiety disorder^52^) represented the comparison group.

Several categories of observed cues were identified in the studies. Most of the studies (*n *= 13, 39.4%; 28, 53, 56, 64, 67–75) focused on heart-related cues, followed by studies focusing on skin-related cues (*n *= 8, 24.2%; 26, 53, 63–68), smartphone usage (*n *= 7, 21.2%; 28, 57–58, 78–79, 81–82), speech and language (*n *= 5, 15.2%; 57–61), activity and motion (*n *= 4, 12.1%; 28, 75, 78–79), location data (*n *= 4, 12.1%; 28, 57, 79–80), sleep (*n *= 3, 9.1%; 28, 57, 77), facial expressions (*n *= 2, 6.1%; 55–56), in-game features (*n *= 2, 6.1%; 83–84), breathing cues (*n *= 1, 3.0%; 62) and cognitive control (*n *= 1, 3.0%; 76). Two-thirds (66.7%; 26, 55, 59–63, 65–66, 69–74, 76–77, 80–84) of studies analysed cues in only one of the categories, while one-third (33.3%; 28, 53, 56–58, 64, 67–68, 75, 78–79) explored cues in multiple categories. The studies reported various methods of detecting these cues (Table 1).

**Table 1.** Summary of the articles included in the review.

Authors, Year, Country	Sample	Anxiety Diagnosis	Comorbidity	Comparison Group	Observed Cues	Method of Observation
Allsop et al. (2017), International	*N* = 16; general adult sample	No (induced anxiety)	No comorbidity	None (high vs low anxiety manipulation condition)	Facial expressions (gaze: % dwell time, scanning entropy, transition frequency), heart-related cues (heart rate)	Eye-tracker software; chest strap
Bauerly & Bilardello (2021), USA	*N* = 28; *N*(adults who stutter) = 13	No (symptoms of social interaction anxiety and fear of negative evaluation assessed with SIAS and BFNE-II, respectively)	No comorbidity	Adults who do not stutter	Skin-related features (skin conductance level), heart-related cues (respiratory sinus arrhythmia)	Biopac MP 160 (Biopac Systems, Inc.)
Berlovskaya et al. (2020), Russia	*N* = 32; student sample	No (symptoms of state and trait anxiety assessed with STAI)	No comorbidity	None	Skin-related features (galvanic skin response; body radiation: intensity of terahertz in the preorbital area and forehead); heart-related cues (heart rate; blood pressure: Kerdo’s index)	Camera system with specific lens; ECG
Boukhechba et al. (2018), USA	*N* = 228; student sample	No (symptoms of social interaction and state anxiety assessed with SIAS, SUDS and BSAM)	No comorbidity	None	Location data (time at home and other locations; transitions between locations)	Smartphone app (GPS data)
Braund et al. (2023), Australia	*N* = 934; secondary school adolescent sample	No (symptoms of anxiety assessed SCAS-S)	No information regarding reported diagnosis, but depressive symptoms present in the sample	None (categorisation of participants based on the SCAS-S score)	Smartphone usage (smartphone keystroke data)	Smartphone
Cao et al. (2022), China	*N* = 80; adult sample	No (symptoms of social anxiety assessed with LSAS)	No comorbidity	None (comparison of high and low social anxiety participants)	Cognitive control (accuracy, reaction time)	EEG, presentation software
Choudhary et al. (2022), International	*N* = 229; general adult sample	No (symptoms of generalised anxiety assessed with GAD)	Self-reported diagnosis of depression in 22% of participants	None (categorisation of participants based on the GAD score)	Smartphone usage (smartphone apps usage and other usage data)	Smartphone
Christian et al. (2023), USA	*N* = 109; student sample	No (symptoms of social interaction and state anxiety assessed with SIAS, SUDS and BSAM)	32.1% participants met clinical threshold for eating disorders	None	Skin-related features (electrodermal activity), heart-related cues (heart-rate variability)	Empatica E4 wristband
Dechant et al. (2021a), no information	*N* = 116; general adult sample	No (symptoms of social anxiety assessed with LSAS)	No information	None (categorisation of anxious and non-anxious participants based on self-report)	In-game features (distance to NPC, path length, time in room)	Computer (in-game)
Dechant et al. (2021b), USA and Canada	*N* = 102; general adult sample	No (symptoms of social anxiety assessed with LSAS)	No comorbidity	None	In-game features (distance to NPC, path length, time in room)	Computer (in-game)
Di Matteo et al. (2021), Canada	*N* = 84; general adult sample	No (symptoms of social and generalised anxiety assessed with LSAS and GAD, respectively)	37% participants were categorised as depressed	None (categorisation of participants between those with positive or negative screening for mental illness)	Speech & language (speech presence; language - use of specific words); sleep (sleep disturbance); location data (location variability, transition between locations), smartphone usage (daily similarity of smartphone usage, screen-related characteristics)	Smartphone
D'Mello et al. (2022), no information	*N* = 475; student sample with elevated stress score	No (symptoms of stress assessed with PSS and generalised anxiety assessed with GAD-7)	No comorbidity	None	Activity and motion (accelerometer values); location data (general location); smartphone usage (screen unlocked time)	mindLAMP app
Dunn et al. (2015), USA	*N* = 240; general adult sample	No (symptoms of social anxiety assessed with SIAS)	No comorbidity	None	Heart-related cues (heart-rate variability: interbeat interval)	Wristwatch and elastic chest band sensor
Fukuda et al. (2020), Japan	*N* = 60; adult sample of office employees	No (symptoms of anxiety assessed with DAMS)	No comorbidity	None	Sleep-related cues (total sleep time, awake events during sleep, deep sleep, light sleep, REM sleep)	Fitbit Charge 3
Gavrilescu and Vizireanu (2019), Romania	*N* = 128; general adult sample	No (symptoms of anxiety assessed with DASS)	Some participants had MD or PTSD, no participant displayed comorbid conditions	None	Facial expressions (specific combination of action units)	Sony PMW-300 One camera with 30 fps and 1024 × 768 resolution
Gong et al. (2019), USA	*N* = 52; student sample	No (symptoms of social anxiety assessed with SIAS)	No comorbidity	None	Activity and motion during smartphone usage; smartphone usage (calls and location, texts and location)	Smartphone
Handouzi et al. (2014), France	*N* = 14; student sample with social anxiety	No (symptoms of social anxiety assessed with LSAS)	No comorbidity	None	Heart-related cues (heart-rate variability, blood volume pulse signal)	Photoplethysmograph device
Jacobson and Feng (2022), USA	*N* = 264; general adult sample	No (symptoms of generalised anxiety assessed with GAD)	No comorbidity	None	Activity and motion (movement intensity); heart-related cues (heart rate, blood pressure)	Actigraphy sensor (ActiGraph - elasticised fabric belt); no information for heart-related cues
Jeppesen et al. (2023), Denmark	*N* = 405; adult patients with suspected lung cancer	No (symptoms of state and trait anxiety assessed with STAI)	Suspected lung cancer	None	Heart-related cues (heart rate, systolic and diastolic blood pressure)	No information
Mauriz et al. (2020), Spain	*N* = 40; student sample	No (symptoms of state and trait anxiety assessed with STAI)	No comorbidity	None	Skin-related features (skin temperature)	Infrared camera
Mori and Haruno (2021), Japan	*N* = 239; general adult sample	No (symptoms of state and trait anxiety assessed with STAI)	No comorbidity	None	Speech & language (length of speech, language - use of specific words)	Twitter posts
Moshe et al. (2021), Europe (mostly Finland)	*N* = 60; general adult sample	No (symptoms of anxiety assessed with DASS)	No comorbidity	None	Heart-related cues (heart rate variability), sleep (time in bed, total sleep time, sleep onset latency, awake events during sleep), activity and motion (number of steps, metabolic equivalent for task), location data (location entropy, time at home, location variability), smartphone usage (frequency, time)	Oura ring, GPS, Delphi app (iOS), AWARE open-source framework
Petrescu et al. (2020), Romania	*N* = 7; general adult sample	No (symptoms of social anxiety assessed with SUDS)	No comorbidity	None	Skin-related features (skin conductance, galvanic skin response)	A wireless sensor (The Shimmer3 GSR+ Unit)
Phitayakorn et al. (2015), USA	*N* = 121; sample of healthcare professionals	No (symptoms of state and trait anxiety assessed with STAI)	No comorbidity	None	Skin-related features (galvanic skin response)	Wristband
Qu et al. (2020), China	*N* = 139, *N*(wristband) = 35; secondary school adolescent sample	No (symptoms of math anxiety assessed with MASC)	No comorbidity	None	Skin-related features (skin conductance), heart-related (heart rate)	Custom designed wristband
Rodrigues et al. 2020), Switzerland	*N* = 135; general young adult sample	No (symptoms of state and trait anxiety assessed with STAI)	No comorbidity	None	Heart-related cues (heart-rate variability)	ECG
Shachter et al. (2022), Japan	*N* = 10; student sample	No (symptoms of anxiety assessed with SAS)	No information	None	Heart-related cues (heart rate)	Smart watch (Fitbit)
Shaukat-Jali et al. (2021), United Kingdom	*N* = 12; young adult sample with subclinical social anxiety	No (symptoms of social anxiety and social phobia assessed with LSAS-SR and SPSQ)	No comorbidity	None	Skin-related features (skin temperature)	Empatica E4 wristband
Suzuki and Sato (2023), Japan	*N* = 28; sample of male students	No (symptoms of state and trait anxiety assessed with STAI)	No comorbidity	None	Breathing cues (exhalation time and variability, inhalation time and variability, exhalation-inhalation features)	Band-type respiration sensor
Tlachac et al. (2021), no information	*N* = 69; general adult sample	No (symptoms of generalised anxiety assessed with GAD)	No comorbidity	None	Speech & language features (length of speech), smartphone usage (apps usage, smartphone communication, other usage data)	EMU mobile data collection app
Weeks et al. (2016), USA	*N* = 28, *N*(social anxiety disorder) = 16; general adult sample and patients with social anxiety disorder	Yes (social anxiety disorder by DSM-IV criteria)	Some participants had comorbidities (no further information)	None	Speech & language features (vocal pitch)	Computerised Speech Lab (CSL), Model 4500 (Kay Elemetrics Corporation 2008)
Xing et al. (2019), China	*N* = 167; general student sample	No (self-declared social phobia)	No comorbidity	None	Heart-related cues (respiratory sinus arrhythmia)	ECG by SOMNOtouch™ RESP
Yu et al. (2023), China	*N* = 1039; general adult sample	No (symptoms of generalised anxiety assessed with SRAS)	No information	None	Speech & language features (language - use of specific words)	Text from Weibo

*Notes*: Abbreviations of measures used to assess anxiety: BFNE: Brief Fear of Negative Evaluation scale; DAMS: Depression and Anxiety Mood Scale; DASS: Depression Anxiety Stress Scale; GAD: Generalised Anxiety Scale; LSAS: Liebowitz Social Anxiety Scale; LSAS-SR: Liebowitz Social Anxiety Scale - Self-Rated; MASC: Multidimensional Anxiety Scale for Children; PSS: Perceived Stress Scale; SAS: State Affect Scale; SCAS-S: Spence Children's Anxiety Scale Short-Form; SIAS: Social Interaction Anxiety Scale; SPSQ: Social Phobia Screening Questionnaire; SRAS: Self-Rating Anxiety Scale; STAI: State-Trait Anxiety Inventory; SUDS: Subjective Units of Distress Scale.

In the following sections, we report the general results of reviewed studies by the main categories. We first explain which observable cues of anxiety were identified in the included studies (RQ1) and, second, describe how the identified studies approached measuring these cues (RQ2). Tables with detailed results for all reported cues and studies are available in the supplemental materials.

### Cues related to facial expressions

In the category of cues related to facial expressions (see Table S1 in the supplemental materials), two cues were observed, in particular specific facial expressions and gaze. A specific combination of action units extracted according to the Facial Action Coding System, a system of taxonomising human facial movements,^53^ was relatively accurate in predicting self-reported anxiety.^54^ Regarding gaze, no significant results relating its specific features to anxiety were observed.^55^

Studies focusing on facial expressions detected those with eye-tracker software capable of processing eye positions and movements,^55^ and the Sony PMW-300 One camera, a camcorder designed for video production.^54^

### Cues related to speech and language

In the category of speech and language cues, four specific groups of cues were identified, namely speech presence, vocal pitch, length of speech and use of specific words (see Table S2 in the supplemental materials). One study^56^ explored speech via smartphone monitoring, with data collected periodically and completely passively throughout 2 weeks of the study; in the case of audio data, one 15-second recording of the ambient audio was performed every 5 minutes. The study found that general speech presence increased the odds of positive screening for social anxiety. Another study^57^ explored the length of speech in recordings submitted by participants during their participation in a survey and found a negative correlation between the length of unscripted speech and self-reported anxiety, meaning that individuals with higher levels of anxiety produced shorter unscripted speech. On the other hand, the same study found a positive correlation between longer scripted speech and anxiety. Similarly, another study that analysed social media data of Twitter users who posted more than 100 tweets prior to participating in the study found that the length of tweets and sentences was positively related to trait anxiety; as such, individuals with higher levels of trait anxiety produced longer tweets and sentences.^58^

One study^59^ analysed audio recordings of semi-structured diagnostic interviews and explored vocal pitch. The authors specifically focused on the mean fundamental frequency, which reflects the rate at which the vocal folds open and close across the glottis during phonation and is the primary factor of our auditory impression of vocal pitch. The study found a positive association between higher vocal pitch and social anxiety disorder, but only for male participants.

Several studies have investigated the use of specific words and their relation to anxiety. Death-related words increased the odds of positive screening for social anxiety.^56^ Moreover, those scoring higher on anxiety measures used a higher proportion of negative and a lower proportion of positive words.^58^

Studies focusing on speech and language cues detected them via smartphone^56^ or, more specifically, via the Early Mental Health Uncovering (EMU) mobile data collection app (i.e., an Android app capable of administering surveys and accessing social media data),^57^ Computerised Speech Lab (CSL) technology (i.e., a hardware and software system for acquisition, analysis, display and playback of speech signals),^59^ posts on Twitter, now known as X^58^ and text from the social media platform Sina Weibo.^60^

### Cues related to breathing

Only one study focused on cues related to breathing.^61^ More specifically, the authors explored whether exhalation and inhalation time, variability and various breathing-related ratios obtained during a 5-minute resting period were associated with state and trait anxiety, measured with a questionnaire (see also Table S3 in the supplemental materials). Among these, only inhalation times and inhalation time variability were significantly positively correlated with trait anxiety, highlighting that individuals with higher levels of anxiety exhibit longer inhalation times and larger inhalation time variability. Breathing cues were observed via a chest-mounted band-type respiration sensor.^61^

### Cues related to the skin

Several reviewed studies explored cues related to skin-type measurements, such as temperature, conductance, electrodermal activity, galvanic skin response and body radiation (see also Table S4 in the supplemental materials). Results on the relationship between skin temperature and anxiety were similar in two studies. Skin temperature proved to be effective in differentiating severity levels of social anxiety^25^ and was positively correlated with self-reported anxiety in several areas of measurement,^62^ meaning that higher skin temperature generally indicates higher levels of anxiety. Similarly consistent are the results regarding electrodermal activity, which was effective in differentiating social anxiety states^25^ and positively associated with social anxiety.^63^ In other words, higher electrodermal activity generally suggests higher levels of anxiety.

Findings on galvanic skin response and skin conductance are less conclusive. Galvanic skin response was positively related to higher levels of social anxiety in one study,^64^ while two other studies^57,58^ found no significant relationship either with trait or state anxiety. The notion that skin conductance is associated with anxiety also received mixed support in reviewed studies. It was positively associated with higher social anxiety^64^ and was higher for adults who stutter compared to those who do not,^52^ while another study^65^ found negative correlations between skin conductance level and anxiety experienced during a math exam.

One study also explored the relationship between body radiation (i.e., indices of electromagnetic radiation in the infrared and terahertz ranges) and anxiety,^66^ but found no significant associations.

Studies focusing on skin-related features detected them via a wristband (Empatica E4 wristband^25,63^; custom designed wristband^65^; or unspecified wristband^67^), a camera system with a specific lens^66^ or infrared camera,^62^ a band-type respiration sensor,^61^ a wireless sensor,^64^ or the Biopac MP 160, a modular data acquisition and analysis system.^52^

### Cues related to the heart

Higher heart rate (see also Table S5 in the supplemental materials) was mainly positively related to higher levels of self-reported anxiety,^55,68^ including anxiety experienced during a math exam.^65^ However, not all studies found a significant relationship between these variables.^66,69^ One study^25^ also reported that heart rate had the lowest effectiveness in predicting anxiety among the examined features, which included heart rate, skin temperature and electrodermal activity. The results pertaining to heart rate variability (i.e., a measure of the variation in time between each heartbeat) are generally similar; studies mostly found significant positive associations between anxiety and higher heart rate variability.^27,63^ However, this was not the case in all studies; Rodrigues and colleagues^70^ did not find any association with their integrated heart rate variation index – a measure of heart rate variability created for their study. Interestingly, one of the studies found a significant interaction between social anxiety and face judgments, where arousal, characterised by the interbeat interval, decreased from baseline to emotion-inducing video for individuals with high social anxiety.^71^

Respiratory sinus arrhythmia (i.e., the cyclic rise and fall of the heart rate in rhythm with breathing) was negatively related to social phobia,^72^ indicating that those with high levels of social phobia exhibited lower respiratory sinus arrhythmia. Respiratory sinus arrhythmia was also lower for adults who stutter than those who do not in the same study.^52^ Moreover, the blood volume pulse signal – another measure of heart rate variability, focused on changes in blood volume – discriminated well between the anxious and calm state.^73^ On the other hand, no significant results were found for the relationship between blood pressure and anxiety.^66,68^

Heart related features were detected via wristbands (Empatica E4 wristband^19,55^; custom designed wristband^65^; wrist/smart watch^69,71^), chest-strap sensors,^55,71^ ECG,^66,70,72^ the Oura smart ring,^27^ the BIOPAC MP 160,^52^ and photoplethysmography,^73^ a simple optical technique used to detect blood volume changes. On the other hand, two studies^68,74^ provided no information on methods of cue detection.

### Cues related to cognitive control

Similarly to the category of cues related to breathing, only one study focused on cues related to cognitive control, which refers to individuals’ ability to direct attention towards task-related information while ignoring irrelevant distractors.^75^ Authors presented participants with the Stroop task, a reliable measure of cognitive control, in which the colour of a word can be congruent (e.g., the word blue written in blue colour) or incongruent with semantic information (e.g., the word purple written in green colour). Participants need to name the colour of the word, and their reaction times are recorded. The study found significantly slower reaction times among participants with high anxiety compared to the comparison group with low anxiety (see Table S6 in the supplemental materials).

### Cues related to sleep

Three reviewed studies focused on cues related to sleep (see Table S7 in supplemental materials). One study^76^ explored several cues, such as time in bed, total sleep time, sleep onset latency (i.e., the time it takes a person to fall asleep), awake events during sleep, deep sleep, light sleep and REM sleep indicators, and classified them according to their importance in predicting anxiety. The authors found that REM/sleep time ratio, REM sleep minutes and light sleep time ratio were the top three sleep-related cues in predicting self-reported anxiety. Another study^56^ found that weeknight sleep disturbance decreased the odds of positive screening for social anxiety. A third study that focused on cues related to sleep^27^ found no significant relationship between total sleep time, sleep onset latency, wake time after sleep onset and anxiety measures.

Sleep-related cues were detected via the Oura smart ring,^27^ Fitbit Charge 3 (i.e., a fitness tracking device)^76^ or a smartphone.^56^

### Cues related to activity and motion

Within the activity- and motion-related cues category, which refer to various aspects of individuals’ movement, authors explored the number of steps, metabolic equivalent of task (i.e., the ratio of active to resting metabolic rate), accelerometer measures, movement intensity and movement during/around calls or texts (see also Table S8 in the supplemental materials). Significant associations with anxiety were first found for movement intensity, whereby higher movement intensity was positively correlated with the severity of generalised anxiety symptoms.^74^ Second, another study found a significant relationship with movement during/around calls or texts, whereby motion dynamics during a phone call in general and at food and leisure locations, but not in other locations or for texts, were correlated with higher social anxiety.^77^

Studies focusing on activity and motion detected those via an actigraphy sensor in the form of an elasticised fabric belt capable of measuring movement intensity,^74^ Oura smart ring and GPS,^27^ smartphone^77^ or the mindLAMP mobile application.^78^

### Cues related to location data

In the category of cues related to location data, the reviewed studies explored the following groups of cues: distance, location entropy (i.e., a measure of the popularity of various locations), time spent at various locations, location variability and transitions between locations (see also Table S9 in the supplemental materials). Time spent at various locations was significantly related to social interaction anxiety in the following instances: a positive correlation of time in food locations at any time of the day (except between 4 pm and 12 am), a positive correlation of time in leisure locations between 8 am and 4 pm, a negative correlation between 4 pm and 8 am, a positive correlation of time in out-of-town locations between 4 pm and 12 am and a positive correlation of time in supermarket at any time of the day.^79^ The same study also found a significant positive correlation between more time at home between 4 pm and 12 am and higher social interaction anxiety, but not at other times,^79^ while another study found no significant associations between time at home and anxiety.^27^

The same two studies also explored location variability and transitions between locations. Regarding location variability, the diversity of places visited was negatively related to social interaction anxiety,^79^ meaning that individuals with higher social interaction anxiety exhibited a lower diversity of places visited. Moreover, a higher number of visited locations was associated with decreased odds of positive social anxiety screening,^56^ while variability in GPS locations exhibited no significant relationship with anxiety.^27^ One study also explored transitions between various locations and found significant positive correlations between higher social interaction anxiety and a higher number of transitions from locations related to education to supermarket locations, from out-of-town to religious locations, from out-of-town to leisure locations and from supermarket to education locations. They also found significant negative correlations between social interaction anxiety and transitions from leisure location to other houses, between two leisure locations and from service to leisure location.^79^ Also, the number of exits from home was associated with decreased odds of positive screening for social anxiety.^56^ Other explored cues related to location data were not significantly associated with anxiety.

Location-related cues were detected via a GPS,^27^ a smartphone app^56,79^ or the mindLAMP mobile application specifically.^78^

### Cues related to smartphone use

Several cues related to smartphone use were explored in relation to anxiety in the reviewed studies (see also Table S10 in the supplemental materials). Daily similarity of smartphone usage (i.e., quantifying the periodic volume of audio recording for indication of regularity of one’s patterns^56^) was associated with decreased odds of positive screening for social anxiety.^56^ There was some mixed support for the association between screen time and self-reported anxiety: higher screen use was associated with increased social anxiety and higher screen time in darkness was associated with decreased odds of positive screening for social anxiety,^56^ while another study found no significant associations between generalised anxiety and time that participants had their smartphone screen unlocked.^78^

Previous studies also found some interesting results regarding smartphone app usage. More time in passive information consumption apps was positively associated with anxiety and differentiated between no anxiety and severe anxiety groups, while the number of times these apps were opened was not a significant differentiator between levels of anxiety. On the other hand, more time in health and fitness apps was negatively related to anxiety and differentiated well between no anxiety and severe anxiety groups, with the latter group exhibiting less time in health and fitness apps.^80^ Some significant associations were also observed between smartphone communication cues and anxiety; a higher number of contacts and a higher number of calls were both positively related to higher anxiety.^26^ Another study found a positive correlation between the percentage of texts and calls at home and anxiety and a negative correlation between the percentage of texts during personal activities and anxiety, while no significant associations were observed in the case of other locations.^77^ Other explored cues, such as smartphone usage frequency and time, keystroke data and the number of texts and tweets, were not significantly related to anxiety.^27,57,80,81^

Smartphone use-related cues were detected via a smartphone (further unspecified),^56,77,80,81^ or specific apps, such as the EMU mobile data collection app,^57^ mindLAMP app^78^ and Delphi data acquisition app.^27^

### Cues related to in-game behaviour

The last identified category of anxiety cues are cues related to in-game behaviour, which stem from the notion that players with social anxiety may display similar behaviours in virtual worlds (i.e., games) as in the physical world. This category of cues was explored in two studies^82,83^ (see Table S11 in Supplemental materials). The most explored cues fall into the group of cues on the distance to non-player characters (i.e., other characters within the game), which in most cases exhibited a positive association with social anxiety, meaning that individuals with higher levels of social anxiety maintained larger distances between their characters and non-player characters. The results for time spent in the 3D game room (i.e., the game environment, built by researchers) from the start to the completion of the trial were mixed; whereas one study found this time to be a significant positive predictor of trait social anxiety,^83^ another found a negative association, but only for avatars (i.e., game characters) that were customised by players in a game played in first-person perspective.^82^ Results regarding the path length (i.e., travelled distance per trial) are similarly mixed. One study found no significant associations between path length and social anxiety.^83^ However, another study found a positive association for predefined avatars (i.e., avatars with generic characteristics) and a negative association for customised avatars (i.e., avatars with the appearance and outfit selected by players), for both first- and third-person perspectives.^82^ In other words, a longer travelled distance per trial was positively associated with higher anxiety when participants could not modify their avatars, and negatively associated with anxiety when participants could customise their own characters in the video game. In-game-related cues were observed via a computer (in-game^82,83^).

## Discussion

As current methods of screening for anxiety disorders employed in healthcare settings do not result in sufficient recognition, anxiety remains significantly underdiagnosed, misdiagnosed and inappropriately treated.^8,29,84^ Therefore, screening could be improved by developing new methods to capture spontaneously generated cues of anxiety by employing digital tools coupled with AI algorithms.^85^ In the present review, we aimed to identify observable cues of anxiety that could be exploited in such technologies, i.e., (RQ1) *What are meaningful observable cues that can offer a valid insight into individuals’ anxiety?*, and identify how are they measured, i.e., (RQ2) *How are those cues measured?*. The results pertaining to RQ1 generally support the idea of observable cues of anxiety, but the findings on specific anxiety cues are somewhat inconclusive. To respond to RQ2, we extracted and summarised information on how observable cues were measured in the reviewed studies.

In this scoping review, we identified 33 studies on observable cues of anxiety published in the last 10 years (i.e., 2014 and onwards). The findings revealed several physiological and behavioural characteristics associated with anxiety disorders categorised in 11 different categories, namely cues related to facial expressions, speech and language, breathing, skin-related cues, cues related to heart, cognitive control, sleep, activity and motion, location data, smartphone use and cues related to in-game behaviour. Even though our review included studies published over a relatively long period of time, we identified only a limited number of studies investigating these phenomena. As a consequence, most of the identified cues were explored only in a single study. When, however, multiple studies analysed the same (or at least similar cues), the findings were, in many cases, mixed. In other words, they implied different relationships between the explored cues and anxiety.

Nevertheless, we extracted several cues of anxiety worth exploring in future studies. In the category of facial expressions, the configuration of specific facial action units may offer insight into individuals’ feelings of anxiety. In the speech and language category, anxiety was related to longer scripted and shorter unscripted speech and longer sentences, higher mean fundamental frequency in male participants, more speech presence and more frequent use of specific words, i.e., death-related words and negative words, and less frequent use of positive words. Among the categories related to physiological responses, longer inhalation and exhalation time (and larger variability), higher skin temperature and higher electrodermal activity received the most uniform support for their relations to anxiety, along with lower respiratory sinus arrhythmia and blood volume pulse signal. Among the cues related to cognitive control, slower reaction times were related to higher anxiety scores. In the categories that refer to behavioural patterns, we identified the following cues: REM/sleep time ratio, REM sleep minutes, light sleep time and sleep disturbances as predictors of anxiety. Also, higher movement intensity and movement during/around calls or texts were related to anxiety, as well as more time spent in specific locations at specific times, lower location variability and fewer transitions between locations. Additional cues were identified in the category of one's behaviour in the digital world, such as distance to non-player characters, time spent in a game room, higher consistency in daily use of smartphones, more time in passive information consumption apps and less time in health and fitness apps, higher number of contacts and higher number of calls. The studies reviewed, therefore, do reveal several cues of anxiety that may be explored further.

Pertaining mostly to cues originating from communicative modalities and those related to physiological responses, authors often utilised computer-aided solutions that extract the values of the cues consistently and independently of the observer to objectively measure the cues. Thus, the quality of features depends only on the precision/resolution of the devices and digital tools, rather than the subjective perception of the observer, which is the case when using self-report or questionnaires. Nevertheless, it is worth mentioning as a response to our RQ2, that observable cues were generally collected via various types of cameras and camera systems with specific software, various wearables, computers and specific software, smartphones with various applications, social media and online platforms, and other sensors.

While we identified several observable cues of anxiety in the review, the topic warrants further attention, especially due to mixed support for many of the cues and a wide variety of technologies used to capture these cues. As this scoping review provides only an overview of the topic,^86^ in our case, an overview of anxiety cues and their ways of measurement, further, more focused reviews may be performed in the future. Such reviews may explore the comparability of methodological approaches employed in the reviewed studies and provide a more detailed comparison of their outcomes. Future studies should, therefore, provide clearer support for cues identified in our review and potentially identify additional, previously undiscovered cues. Additionally, as has already been observed in the previous study on cues of depression,^87^ this review has revealed that many studies focused only on a single modality or category when exploring potential cues of anxiety, while it is very likely that anxiety is concurrently expressed via several of them. This notion is important both for future studies exploring the identified cues of anxiety as well as for developing new tools for their detection. In fact, previous studies have shown that when developing AI algorithms for the recognition of mental health disorders, such as depression, unimodal approaches perform less optimally than multi-modal approaches,^88,89^ which should also be explored in the context of anxiety recognition. Therefore, future studies should focus on developing additional pathways that could provide further insight and aid in overcoming current barriers and would also allow for an early diagnosis that would significantly enhance the health, function and well-being of patients.^31^ Such aids for establishing diagnosis could potentially be incorporated into patients’ consultations with their healthcare providers for screening of cues linked to mental health disorders or even used for continuous monitoring indicated for at-risk individuals, be it at healthcare facilities or even remotely.

### Limitations

While this scoping review offers a valuable synthesis of research on the observable cues of anxiety disorders and how they are measured, we need to mention some limitations. As indicated by the review, there is no set of features focusing on anxiety in general or discussing the moderators of how the cues are presented and to what extent they depend on the level of specific disorder. Thus, in other words, future research should explore which cues can be generalised and which of them are relevant in which cases to allow the development of algorithms robust to the highly individual nature of how anxiety disorders are expressed. Additionally, since we focused only on English-language papers based on studies that were largely conducted on Anglophone participants in industrialised countries, our conclusions may be culturally biased. Similarly, our findings are limited by the fact that the majority of included studies were cross-sectional. Further research is thus needed to understand whether our findings can be generalised. While not very likely (as studies in this field skyrocketed in recent years), it is also possible that we overlooked some relevant studies due to limiting ourselves to articles published in the last 10 years. Lastly, it is worth noting that we conducted a broad scoping review following established methodology^90^ with the aim of conducting a complete overview of the research activity related to our research question and hence did not evaluate the quality of included articles; future research may investigate more specific research questions and conduct a risk of bias assessment.

## Conclusions

In this research, we analysed the relevant literature to synthesise a set of cues that could be used to screen for anxiety. We focused on cues that are generated spontaneously, in individuals’ natural environment, and can be measured without the use of any specialised equipment non-accessible to general consumers. The review led us to a plethora of cues that show a high potential for screening for anxiety. As such, an important theoretical contribution of our study is a comprehensive framework of anxiety that takes into account its multifaceted nature and provides theoretical underpinnings for the development of future technologies. Moreover, as pointed out by our scoping review, we identified several cues where further research is needed, and some shortcomings in the literature, such as a significant lack of available prospective cohorts, providing valuable guidance for future research in this area. On the more practical side, we argue that our review may help harness the potential of future developments, such as AI tools, in detecting and managing anxiety. Such tools may improve the accuracy and effectiveness of the existing screening and diagnostic methods, with a particular advantage being the possibility to continuously monitor individuals and develop systems for early detection of warning signs, allowing for timely intervention and prevention of more severe symptoms. Furthermore, our findings could inform the design of everyday technology, such as smartphones, to include features that monitor and respond to anxiety cues. This integration may make mental health support more accessible, discreet and user-friendly, encouraging individuals to seek help without stigma.

## Supplemental Material

sj-docx-1-dhj-10.1177_20552076241297006 - Supplemental material for Decoding anxiety: A scoping review of observable cues Supplemental material, sj-docx-1-dhj-10.1177_20552076241297006 for Decoding anxiety: A scoping review of observable cues by Urška Smrke, Izidor Mlakar, Ana Rehberger, Leon Žužek and Nejc Plohl in DIGITAL HEALTH

## Notes

1. Despite its classification as an anxiety disorder in DSM-V, studies about selective mutism were excluded as individuals with this disorder might not exhibit typical observable, in particular verbal, cues of anxiety. This was done to avoid introducing variability that might confound our findings and make it more difficult to generalise them.
