# Supplementary material for: Decoding anxiety: A scoping review of observable cues
Source: Digit Health. 2024 Nov 26;10:20552076241297006. doi: 10.1177/20552076241297006 (PMC11590141; doi:10.1177/20552076241297006)
Supplement: sj-docx-1-dhj-10.1177_20552076241297006 - Supplemental material for Decoding anxiety: A scoping review of observable cues [file sj-docx-1-dhj-10.1177_20552076241297006.docx]

**Supplemental materials**

Table S1. Facial expressions related observable cues from the articles included in the review.

| **Group of observed cues** | **Specific observed cues** | **Relation of cue to anxiety** | **Authors, year** |
| --- | --- | --- | --- |
| Specific facial expressions | Combination of action units AU2, AU9, AU25, AU45 | 72.9 % accuracy in predicting anxiety | Gavrilescu & Vizireanu (2019) |
| Gaze | % dwell time | No sig. main effect for anxiety (F(1,15) = 4.15, η2 = 0.22) | Allsop et al. (2017) |
|  | Scanning entropy | No sig. main effect for anxiety (F(1,15) = 0.30, η2 = 0.02) | Allsop et al. (2017) |
|  | Transition frequency | No sig. main effect for anxiety (F(1,15) = 0.05, η2 = 0.003) | Allsop et al. (2017) |

*Notes.* Where significance of the results is not reported (by sig. or non sig. description), the information was not available in the reviewed paper.

­

Table S2. Speech and language related observable cues from the articles included in the review.

| **Group of observed cues** | **Specific observed cues** | **Relation of cue to anxiety** | **Authors, year** |
| --- | --- | --- | --- |
| Speech presence | Speech presence | Increased odds of positive screening for social anxiety (OR =~ 0.10) | Di Matteo et al. (2021) |
| Vocal pitch | Mean fundamental frequency (F0) | Positive association with social anxiety disorder (F = 6.07, partial η2 = .43) and sig. positive association during the initiation of the interviews in males (F = 16.59*) but no sig. association in females (all Fs < 0.76, all partial η2s < .14) | Weeks et al. (2016) |
|  |  | Sig. positive correlation with social anxiety in males (r = 0.57*); successful replication of cut-off for classifying men with and without social anxiety disorder (cut-off: 122.78 Hz, sensitivity: 89%, specificity: 100%) | Weeks et al. (2016) |
| Length of speech | Length of scripted speech | Positive correlation with anxiety (r =~0.30) | Tlachac et al. (2021) |
|  | Length of unscripted speech | Negative correlation with anxiety (r =~ -0.10) | Tlachac et al. (2021) |
|  | Length of tweets and sentences | Sig. positive correlation with trait anxiety (r = 0.22*) | Mori & Haruno (2021) |
| Language - use of specific words | Death related words | Increased odds of positive screening for social anxiety (OR =~ 0.50) | Di Matteo et al. (2021) |
|  | Word categories related to body and mind (e.g., 'health', 'body', 'feel') | No information | Yu et al. (2023) |
|  | First person singular pronouns | No information | Yu et al. (2023) |
|  | Word categories related to negative emotions (e.g., 'sad', 'anx*') | No information | Yu et al. (2023) |
|  | Word categories related to daily work and life (e.g., 'work', 'money', 'leisure', 'social', 'achieve') | No information | Yu et al. (2023) |
|  | Proportion of negative words | Sig. positive correlation with trait anxiety (r =~ 0.15*) | Mori & Haruno (2021) |
|  | Proportion of positive words | Sig. negative correlation with trait anxiety (r =~ -0.15*) | Mori & Haruno (2021) |

*Notes.* Where significance of the results is not reported (by sig. or non sig. description), the information was not available in the reviewed paper.

Table S3. Breathing related observable cues from the articles included in the review.

| **Group of observed cues** | **Specific observed cues** | **Relation of cue to anxiety** | **Authors, year** |
| --- | --- | --- | --- |
| Exhalation time and variability | Exhalation time | Non sig. negative correlation with state anxiety (r = -0.16) and positive correlation with trait anxiety (r = 0.14) | Suzuki & Sato (2023) |
|  | Exhalation time variability | Non sig. negative correlation with state anxiety (r = -0.31) and positive correlation with trait anxiety (r = 0.14) | Suzuki & Sato (2023) |
| Inhalation time and variability | Inhalation time | Non sig. negative correlation with state anxiety (r = -0.21), sig. positive correlation with trait anxiety (r = 0.37*) | Suzuki & Sato (2023) |
|  | Inhalation time variability | Non sig. negative correlation with state anxiety (r = -0.25), sig. positive correlation with trait anxiety (r = 0.40*) | Suzuki & Sato (2023) |
| Exhalation-inhalation ratio, ratio variability, respiratory rate | Exhalation-inhalation ratio | No sig. correlation with state (r = 0.02) nor trait anxiety (r = -0.14) | Suzuki & Sato (2023) |
|  | Exhalation-inhalation ratio variability | No sig. correlation with state (r = -0.06) nor trait anxiety (r = 0.03) | Suzuki & Sato (2023) |
|  | Respiratory rate | No sig. correlation with state (r = 0.11) nor trait anxiety (r = -0.08) | Suzuki & Sato (2023) |

Table S4. Skin related observable cues from the articles included in the review.

| **Group of observed cues** | **Specific observed cues** | **Relation of cue to anxiety** | **Authors, year** |
| --- | --- | --- | --- |
| Skin temperature | Skin temperature | Effective in differentiating severity levels of social anxiety (no further information reported) | Shaukat-Jali et al. (2021) |
|  | Skin temperature | (Sig.) positive correlations with anxiety (forehead skin temperature (ST): r = 0.58*; periorbital SD: r = 0.39*; maxillary ST: r = 0.33*; neck areas ST: r = 0.28) | Mauriz et al. (2020) |
| Skin conductance | Skin conductance level | (Sig.) negative correlations with math evaluation anxiety (at -4min: r = -0.45*, at -3min: r = -0.48*; at -2min: r = -0.40; at -1min: r = -0.44*; at 0min: r = -0.38*) | Qu et al. (2020) |
|  | Skin conductance level | Sig. higher for adults who stutter than those who don't (t(24) = 2.41*); no sig. effect of anxiety on skin conductance level in general (SIAS: beta = 0.17; BFNE-II: beta = 0.03), for adults who stutter (SIAS: beta = 0.01; BFNE-II: beta = -0.13), and those who do not (SIAS: beta = 0.18; BFNE-II: beta = 0.11) | Bauerly & Bilardello (2021) |
|  | Skin conductance level - mean absolute value relative to baseline | Sig. positive correlation with social anxiety (r = 0.72*) | Petrescu et al. (2020) |
|  | Skin conductance level - mean absolute value | Sig. positive correlation with social anxiety (r = 0.35*) | Petrescu et al. (2020) |
|  | Skin conductance level - Willison amplitude | Sig. positive correlation with social anxiety (r = 0.34*) | Petrescu et al. (2020) |
| Electrodermal activity | Electrodermal activity | Effective in differentiating between baseline and social anxiety states (no further information reported) | Shaukat-Jali et al. (2021) |
|  | Electrodermal activity - average peak height | Positive association with trait (SHAP = 0.01) and state (SHAP = 0.09) social anxiety disorder symptoms | Christian et al. (2023) |
|  | Electrodermal activity - maximum peak height | Positive association with trait (SHAP = 0.04) and state (SHAP = 0.1) social anxiety disorder symptoms | Christian et al. (2023) |
|  | Electrodermal activity - number of peaks | Positive association with state social anxiety disorder symptoms (SHAP = 0.07) | Christian et al. (2023) |
| Galvanic skin response | Galvanic skin response | Non sig. positive correlation with state (r = 0.08) and trait (r = 0.18) anxiety | Berlovskaya et al. (2020) |
|  | Galvanic skin response - max | Non sig. correlation with state (r = 0.02) nor trait anxiety (r = -0.11) | Phitayakorn et al. (2015) |
|  | Galvanic skin response - mean | Non sig. correlation with state (r = -0.01) or trait (r = -0.09) anxiety | Phitayakorn et al. (2015) |
|  | Galvanic skin response - mean absolute value relative to baseline | Sig. positive correlation with social anxiety (r = 0.37*) | Petrescu et al. (2020) |
|  | Galvanic skin response - mean absolute value | Sig. positive correlation with social anxiety (r = 0.35*) | Petrescu et al. (2020) |
|  | Galvanic skin response - Willison amplitude | Sig. positive correlation with social anxiety (r = 0.37*) | Petrescu et al. (2020) |
| Body radiation | Intensity of tetraherc radiation in the preorbital area | Non sig. negative correlation with state (r = -0.15) and trait (r = -0.15) anxiety | Berlovskaya et al. (2020) |
|  | Intensity of tetraherc radiradiation on the forehead | Non sig. positive correlation with state (r = 0.02) and trait (r = 0.27) anxiety | Berlovskaya et al. (2020) |

*Notes.* Where significance of the results is not reported (by sig. or non sig. description), the information was not available in the reviewed paper.

Table S5. Heart related observable cues from the articles included in the review.

| **Group of observed cues** | **Specific observed cues** | **Relation of cue to anxiety** | **Authors, year** |
| --- | --- | --- | --- |
| Heart rate | Heart rate | Lowest effectiveness in prediction of anxiety among examined features (no further information reported) | Shaukat-Jali et al. (2021) |
|  | Heart rate | Non sig. positive correlation with state (r = 0.10) and trait (r = 0.10) anxiety) | Berlovskaya et al. (2020) |
|  | Heart rate | Sig. positive correlation (r = 0.23*) and predictor (beta = 0.13*) of anxiety | Jeppesen et al. (2023) |
|  | Heart rate | Non sig. positive correlation with feeling anxious (r = 0.54) and with feeling nervous (r = 0.59) | Shachter et al. (2022) |
|  | Heart rate - time cluster 2 | (Sig.) positive correlation with math evaluation anxiety (r = 0.34 at 63min; r = 0.47* at 64min; r = 0.51* at 65min of exam) | Qu et al. (2020) |
|  | Heart rate - time cluster 3 | (Sig.) positive correlation with math evaluation anxiety (r = 0.53* at 82min; r = 0.41 83min; r = 0.48* at 84min; r =0.54* at 85min; r = 0.45* at 86min of exam) | Qu et al. (2020) |
|  | Heart rate | Sig. main effect for anxiety condition (F(1,15) = 18.07*, η2 = 0.55) | Allsop et al. (2017) |
| Heart rate variability | Heart rate variability | Positive association with trait social anxiety disorder symptoms (SHAP = 0.10) | Christian et al. (2023) |
|  | Heart rate variability | Non sig. positive correlation (r = 0.12) and sig. positive predictor (beta = 0.26*) of anxiety | Moshe et al. (2021) |
|  | Integrated heart rate variation index | No sig. correlation with anxiety (coefficient not reported) | Rodrigues et al. (2020) |
|  |  |  |  |
|  | Interbeat interval | Sig. interaction between social anxiety and Chernoff face judgements – decrease in arousal from baseline to emotion inducing video for individuals with high social anxiety (Wald’s  χ2=7.479, b=-.458, SE=.168, p=.006) | Dunn et al. (2015) |
| Respiratory sinus arrythmia | Respiratory sinus arrythmia | Sig. lower for adults who stutter than those who don't (t(23) = -2.23*); (sig.) negative effect of anxiety in general (SIAS: beta = -0.06*; BFNE-II: beta = -0.04) and for adults who stutter (SIAS: beta = -0.05*; BNFE-II: beta = -0.06*), but no sig. effect of anxiety for adults who do not stutter (SIAS: beta = -0.02, BNFE-II: beta = 0.03) | Bauerly & Bilardello (2021) |
|  | Respiratory sinus arrythmia | Sig. negative correlation with social phobia (r = -0.16*) | Xing et al. (2019) |
| Blood volume pulse signal | Moving average interbeat interval | Discrimination of anxiety and calm state with classification rate of 80% | Handouzi et al. (2014) |
|  | Moving average of blood volume pulse peak-to-peak amplitude variation | Discrimination of anxiety and calm state with classification rate of 93.73% | Handouzi et al. (2014) |
|  | Power spectral density of high frequencies | Discrimination of anxiety and calm state with classification rate of 72.94% | Handouzi et al. (2014) |
| Blood pressure | Systolic blood pressure | No sig. effect for anxiety (beta = 0.07) | Jeppesen et al. (2023) |
|  | Diastolic blood pressure | No sig. effect for anxiety (beta = 0.01) | Jeppesen et al. (2023) |
|  | Kerdo index | No sig. correlation with state (r = -0.02) nor trait anxiety (r = 0.03) | Berlovskaya et al. (2020) |

*Notes.* Where significance of the results is not reported (by sig. or non sig. description), the information was not available in the reviewed paper.

Table S6. Cognitive control related observable cues from the articles included in the review.

| **Group of observed cues** | **Specific observed cues** | **Relation of cue to anxiety** | **Authors, year** |
| --- | --- | --- | --- |
| Accuracy | Accuracy in Stroop taks | No sig. main effect of anxiety (coefficient not reported) | Cao et al. (2022) |
| Reaction time | Reaction time in Stroop task | Sig. main effect of anxiety (F(1,78)=5.765*, partial η2 = 0.069; i.e., slower reaction time in high vs low anxiety group) | Cao et al. (2022) |

Table S7. Sleep related observable cues from the articles included in the review.

| **Group of observed cues** | **Specific observed cues** | **Relation of cue to anxiety** | **Authors, year** |
| --- | --- | --- | --- |
| Time in bed | Time in bed | No sig. correlation (r = 0.03) nor prediction (beta = 0.19) of anxiety | Moshe et al. (2021) |
| Total sleep time | Total sleep minutes | 6th most important feature in predicting anxiety (importance = 0.08) among 13 sleep features examined | Fukuda et al. (2020) |
|  | Total sleep time | No sig. correlation (r = 0.04) nor prediction (beta = 0.13) of anxiety | Moshe et al. (2021) |
| Sleep onset latency | Sleep onset latency | No sig. correlation (r = 0.06) nor prediction (beta = 0.01) of anxiety | Moshe et al. (2021) |
| Awake events during sleep | Wake after sleep onset | No sig. correlation (r = 0.14) not prediction (beta = 0.23) of anxiety | Moshe et al. (2021) |
|  | Number of wake events | 8th most important feature in predicting anxiety (importance = 0.07) among 13 sleep features examined | Fukuda et al. (2020) |
|  | Total wake minutes | 12th most important feature in predicting anxiety (importance = 0.06) among 13 sleep features examined | Fukuda et al. (2020) |
|  | Total wake time ratio | 5th most important feature in predicting anxiety (importance = 0.08) among 13 sleep features examined | Fukuda et al. (2020) |
| Deep sleep | Number of deep sleep events | 11th most important feature in predicting anxiety (importance = 0.04) among 13 sleep features examined | Fukuda et al. (2020) |
|  | Deep sleep minutes | 9th most important feature in predicting anxiety (importance = 0.07) among 13 sleep features examined | Fukuda et al. (2020) |
|  | Deep sleep time ratio | 8th most important feature in predicting anxiety (importance = 0.08) among 13 sleep features examined | Fukuda et al. (2020) |
| Light sleep | Light sleep minutes | 7th most important feature in predicting anxiety (importance = 0.08) among 13 sleep features examined | Fukuda et al. (2020) |
|  | Light sleep time ratio | 3th most important feature in predicting anxiety (importance = 0.09) among 13 sleep features examined | Fukuda et al. (2020) |
|  | Number of light sleep events | 11th most important feature in predicting anxiety (importance = 0.06) among 13 sleep features examined | Fukuda et al. (2020) |
| REM sleep | Number of REM sleep events | 10th most important feature in predicting anxiety (importance = 0.07) among 13 sleep features examined | Fukuda et al. (2020) |
|  | REM sleep minutes | 2nd most important feature in predicting anxiety (importance = 0.10) among 13 sleep features examined | Fukuda et al. (2020) |
|  | REM sleep time ratio | Most important feature in predicting anxiety (importance = 0.11) among 13 sleep features examined | Fukuda et al. (2020) |
| Sleep disturbance | Weeknight sleep disturbance | Decreased odds of positive screening for social anxiety (OR =~ -0.25) | Di Matteo et al. (2021) |

*Notes.* Where significance of the results is not reported (by sig. or non sig. description), the information was not available in the reviewed paper.

Table S8. Activity and motion related observable cues from the articles included in the review.

| **Group of observed cues** | **Specific observed cues** | **Relation of cue to anxiety** | **Authors, year** |
| --- | --- | --- | --- |
| Number of steps | Number of steps | No sig. correlation (r = -0.16) nor prediction (beta = -0.09) of anxiety | Moshe et al. (2021) |
| Metabolic equivalent for task | Metabolic equivalent for task (MET) | No sig. correlation (r = -0.07) nor prediction (beta = -0.09) of anxiety | Moshe et al. (2021) |
| Accelerometer measures | Accelerometter values | Non sig. positive correlation with generalized anxiety (r = 0.09) | D'Mello et al. (2022) |
| Movement intensity | Movement intensity | Positive correlation (r = 0.51, CI (0.42, 0.69)) with severity of generalized anxiety symptoms | Jacobson & Feng (2022) |
| Movement during/around calls / texts | Motion around a call | Sig. positive correlation of motion dynamics of phone call with social anxiety (mean of distances: r = 0.29*; SD of distances: r = 0.30*) | Gong et al. (2019) |
|  | Motion around a call at a location | Sig. positive correlation of motion variation during a phone call at food and leisure location with social anxiety (mean of distances: r = 0.37*; SD of distances: r = 0.45*); no sig. correlation of motion variation at other locations (mean of distances: r = -0.12 (work), r = 0.11 (home), r = -0.33 (personal life), r = 0.22 (transition); SD of distances: r = 0.05 (work), r = 0.21 (home), r = -0.35 (personal life), r = 0.25 (transition)) | Gong et al. (2019) |
|  | Motion around a text | No sig. correlation with social anxiety (mean distance: r = 0.20; SD of distance: r = 0.23) | Gong et al. (2019) |
|  | Motion around a text at a location | No sig. correlation with social anxiety at any location (mean of distance: r = -0.21 (work), r = 0.16 (home), r = 0.16 (food & leisure), r = -0.05 (personal life), r = 0.14 (transition); SD of distances: r = -0.12 (work), r = 0.22 (home), r = 0.22 (food & leisure), r = -0.03 (personal life), r = 0.14 (transition)) | Gong et al. (2019) |

*Notes.* Where significance of the results is not reported (by sig. or non sig. description), the information was not available in the reviewed paper.

Table S9. Location data related observable cues from the articles included in the review.

| **Group of observed cues** | **Specific observed cues** | **Relation of cue to anxiety** | **Authors, year** |
| --- | --- | --- | --- |
| General | Location; (latitude–longitude pairs) | Sig . positive correlation with generalized anxiety (r = 0.19*) | D'Mello et al. (2022) |
| Distance | Total distance | No sig. correlation (r = -0.28) nor prediction (beta = -0.07) of anxiety | Moshe et al. (2021) |
| Location entropy | Location entropy | No sig. correlation (r = -0.17) nor prediction (beta = -0.07) of anxiety | Moshe et al. (2021) |
|  | Location entropy - normalized | No sig. correlation (r = -0.13) nor prediction (beta = -0.00) of anxiety | Moshe et al. (2021) |
| Time at home | Time at home | No sig. correlation (r = 0.13) nor prediction (beta = 0.10) of anxiety | Moshe et al. (2021) |
|  | Time at home | Sig. positive correlation of time at home 4pm-12am with social interaction anxiety (r =~ 0.50*), but no sig. correlation of time at home at any other time (r =~ -0.15 - 0.05 for all day, 8am-4pm, 12am-8am) | Boukhechba et al. (2018) |
| Time at other locations | Time at other houses | No sig. correlation with social interaction anxiety at any time of day (r =~ -0.40 - 0.15 for all day, 8am-4pm, 4pm-12am, 12am-8am) | Boukhechba et al. (2018) |
|  | Time in food locations | Sig. negative correlation with social interaction anxiety at any time of day (r =~ -0.40* for all day, 8am-4pm, 12am-8am), except non sig. correlation for 4pm-12am (r =~ 0.15) | Boukhechba et al. (2018) |
|  | Time in leisure locations | Sig. positive correlation with social interaction anxiety for 8am-4pm (r =~ 0.35*), sig. negative correlation for 4pm-12am (r =~ -0.65*) and 12am-8am (r =~ -0.50*) , no sig. correlation with for all day (r =~ 0.05) | Boukhechba et al. (2018) |
|  | Time in out-of-town locations | Sig. positive correlation with social interaction anxiety for 4pm-12am (r =~ 0.20*), but not for other times (r =~ 0.10 - 0.15 for all day, 8am-4pm, 12am-8am) | Boukhechba et al. (2018) |
|  | Time in religious locations | No sig. correlation with social interaction anxiety at any time of day (r =~ 0.05 - 0.15 for all day, 8am-4pm, 4pm-12am, 12am-8am) | Boukhechba et al. (2018) |
|  | Time in service locations | No sig. correlation with social interaction anxiety at any time of day (r =~ -0.20 - -0.10 for all day, 8am-4pm, 4pm-12am, 12am-8am) | Boukhechba et al. (2018) |
|  | Time in supermarket | (Sig.) positive correlation with social interaction anxiety at any time of day (r =~ 0.50* for all day, r =~ 0.40* for 8am-4pm, r =~ 0.10 for 4pm-12am, r =~ 0.70* for 12am-8am) | Boukhechba et al. (2018) |
| Location variability | Variability in GPS locations | No sig. correlation (r = -0.26) nor prediction (beta = -0.16) of anxiety | Moshe et al. (2021) |
|  | Diversity of places visited | Sig. negative correlation with social interaction anxiety (r = -0.64* on weekdays, r = -0.57* on weekends) | Boukhechba et al. (2018) |
|  | Number of locations visited | Decreased odds of positive screening for social anxiety (OR =~ -0.90) | Di Matteo et al. (2021) |
| Transition between locations | Transition from education to supermarket location | Sig. positive correlation with social interaction anxiety (r = 0.22*) | Boukhechba et al. (2018) |
|  | Transition from leisure location to other houses | Sig. negative correlation with social interaction anxiety (r = -0.22*) | Boukhechba et al. (2018) |
|  | Transition from leisure to leisure location | Sig. negative correlation with social interaction anxiety (r = -0.50*) | Boukhechba et al. (2018) |
|  | Transition from out of town location to religious location | Sig. positive correlation with social interaction anxiety (r = 0.34*) | Boukhechba et al. (2018) |
|  | Transition from out of town to leisure location | Sig. positive correlation with social interaction anxiety (r = 0.28*) | Boukhechba et al. (2018) |
|  | Transition from service to leisure location | Sig. negative correlation with social interaction anxiety (r = -0.29*) | Boukhechba et al. (2018) |
|  | Transition from supermarket to education location | Sig. positive correlation with social interaction anxiety (r = 0.34*) | Boukhechba et al. (2018) |
|  | Number of exits from home | Decreased odds of positive screening for social anxiety (OR =~ -0.15) | Di Matteo et al. (2021) |

*Notes.* Where significance of the results is not reported (by sig. or non sig. description), the information was not available in the reviewed paper.

Table S10. Smartphone use related observable cues from the articles included in the review.

| **Group of observed cues** | **Specific observed cues** | **Relation of cue to anxiety** | **Authors, year** |
| --- | --- | --- | --- |
| Smartphone usage frequency | Usage frequency | No sig. correlation (r = 0.24) nor sig. prediction (beta = 0.14) of anxiety | Moshe et al. (2021) |
| Smartphone usage time | Usage time | No sig. correlation (r = 0.05) nor sig. prediction (beta = 0.06) of anxiety | Moshe et al. (2021) |
| Daily similarity of smartphone usage | Daily similarity of smartphone usage | Decreased odds of positive screening for social anxiety (OR =~ -0.40) | Di Matteo et al. (2021) |
| Other usage data | Other smartphone usage data | Not among three most important features in relation to anxiety any of the tested models and were not further explored (no coefficients reported) | Choudhary et al. (2022) |
|  | Number of events [*the feature is not explained] | Non sig. positive correlation (r =~ 0.20) with anxiety | Tlachac et al. (2021) |
| Screen | Screen unlocked time | Non sig. positive correlation with generalized anxiety (r = 0.15) | D'Mello et al. (2022) |
|  | Screen use | Increased odds of positive screening for social anxiety (OR =~ 0.10) | Di Matteo et al. (2021) |
|  | Screen time in darkness | Decreased odds of positive screening for social anxiety (OR =~ -0.30) | Di Matteo et al. (2021) |
| Smartphone keystroke data | Down down (i.e., the time interval between key press of a keystroke and key press of the following keystroke) | Non sig. negative correlation with anxiety (r = -0.05) | Braund et al. (2023) |
|  | Dwell (i.e., the time interval between a key press and release of the same key) | Non sig. negative correlation with anxiety (r = -0.04) | Braund et al. (2023) |
|  | Interval (i.e., the time interval between a key press of a keystroke and key release of the following key) | Non sig. negative correlation with anxiety (r = -0.02) | Braund et al. (2023) |
|  | Latency (i.e., the time interval between a key press of a keystroke and key release of the following keystroke) | Non sig. negative correlation with anxiety (r = -0.04) | Braund et al. (2023) |
|  | Total backspaces | Non sig. positive correlation with anxiety (r = 0.04) | Braund et al. (2023) |
|  | Total keystrokes | Non sig. correlation with anxiety (r = 0.01) | Braund et al. (2023) |
|  | Total non-alphanumerics | Non sig. correlation with anxiety (r = -0.01) | Braund et al. (2023) |
|  | Total null keys (i.e., unlabeled or undetermined keys) | Non sig. correlation with anxiety (r = -0.04) | Braund et al. (2023) |
|  | Total spaces | Non sig. correlation with anxiety (r = 0.01) | Braund et al. (2023) |
|  | Up up (the time interval between the key release of a keystroke and key release of the following keystroke) | Non sig. correlation with anxiety (r = -0.03) | Braund et al. (2023) |
| Smartphone apps usage | Number of times game apps were opened with session lengths greater than 1 SD from the mean | Third most important feature in the multiclass model of anxiety (score: ~.07), but no sig. differentiation between levels of anxiety (F = 60.22); not among three most important features in the binary model (not explored further) | Choudhary et al. (2022) |
|  | Number of times passive information consumption apps were opened within the 24-hour period | Cue with highest feature importance score in the multiclass model (score: ~0.25) but no sig. differentiation among levels of anxiety (F = 63.40); third most important feature in the binary model (score: ~.08) but no sig. differentiation between no anxiety and severe anxiety groups (d = 0.04) | Choudhary et al. (2022) |
|  | Mean session time within a 24-hour period in "passive information consumption" apps | Second most important feature in the multiclass model (score: ~ .07), sig. differentiation among levels of anxiety (F = 5.23); most important feature in the binary model (score: ~.10) and sig. differentiation between no anxiety and severe anxiety groups (d = 0.18; i.e., shorter average session time in the severe anxiety group) | Choudhary et al. (2022) |
|  | Mean session time within a 24-hour period in the "health and fitness" apps | Not among most important features in the multiclass model (not explored further); second most important feature in binary model and sig. differentiation between no anxiety and severe anxiety groups (d = -0.16; i.e., higher average session time in the severe anxiety group) | Choudhary et al. (2022) |
| Smartphone apps usage - Twitter | Number of Tweets | Non sig. correlation with anxiety (r =~ -0.15) | Tlachac et al. (2021) |
| Smartphone communication - number of contacts | Number of contacts | Positive correlation with anxiety (r =~ 0.30) | Tlachac et al. (2021) |
| Smartphone communication - number of calls | Number of calls | Positive correlation with anxiety (r =~ 0.30) | Tlachac et al. (2021) |
| Calls and location | Percentage of calls at a location | Sig. positive correlation with anxiety at home (r = 0.40*), but not at other locations (r = -0.18 (work), r = -0.23 (food & leisure), r = 0.12 (personal life), r = -0.07 (transition)) | Gong et al. (2019) |
| Text messages | Number of Texts | No correlation with anxiety (r =~ 0.00) | Tlachac et al. (2021) |
| Texts and location | Percentage of texts at a location | Sig. positive correlation with anxiety at home (r = 0.41*) and sig. negative correlation during personal activities (r = -0.29*), but no sig. correlation at other locations (r = -0.25 (work), r = -0.09 (food & leisure), r = -0.07 (transition)) | Gong et al. (2019) |

*Notes.* Where significance of the results is not reported (by sig. or non sig. description), the information was not available in the reviewed paper.

Table S11. In-game related observable cues from the articles included in the review.

| **Group of observed cues** | **Specific observed cues** | **Relation of cue to anxiety** | **Authors, year** |
| --- | --- | --- | --- |
| **Distance to NPC** | Distance to NPC at the end of the task | Sig. positive relation with social anxiety in third-person perspective (beta = 0.19* (customized avatar), beta = 0.30* (predefined avatar)), but no sig. relation in first-person perspective (beta = -0.9 (customized avatar), beta = 0.01 (predefined avatar)) | Dechant et al. (2021a) |
|  | Minimum distance to NPC | Sig. positive relation with social anxiety in first- (beta = 0.19*) and third-person perspective (beta = 0.28*) for custumized avatar, but no sig. relation for predefined avatar (beta = -0.03 (first-person perspective), beta = -0.03 (third-person perspective)) | Dechant et al. (2021a) |
|  | Minimal comfortable distance to the NPC | Sig. positive predictor of trait social anxiety (beta = 0.20*) | Dechant et al. (2021b) |
|  | Mean distance to NPC | Sig. positive relation with social anxiety in first- (beta = 0.16*) and third-person perspective (beta = 0.24*) for custumized avatar, but no sig. relation for predefined avatar (beta = 0.00 (first-person perspective), beta = 0.04 (third-person perspective)) | Dechant et al. (2021a) |
|  | Mean distance to NPC - implicit | Non sig. predictor of trait social anxiety (beta = 0.07) | Dechant et al. (2021b) |
|  | Minimal distance to NPC | Sig. positive predictor of trait social anxiety (beta = 0.27*) | Dechant et al. (2021b) |
|  | Kurtosis of the distribution of distances per trial | Sig. positive relation with social anxiety in first- (beta = 0.13*) and third-person perspective (beta = 0.13*) for custumized avatar, but no sig. relation for predefined avatar (beta = -0.04 (first-person perspective), beta = -0.12 (third-person perspective)) | Dechant et al. (2021a) |
|  | Kurtosis of distances from NPC - implicit | Sig. positive predictor of trait social anxiety (beta = 0.22*) | Dechant et al. (2021b) |
|  | Skew of distances from NPC - implicit | Sig. positive predictor of trait social anxiety (beta = 0.24*) | Dechant et al. (2021b) |
|  | Skew of the distribution of distances per trial | Sig. positive relation with social anxiety with customized avatar in third-person perspective (beta = 0.26*), but no sig. correlation in other conditions (r = -0.11 customized avatar in first-person perspective; predefined avatar: r = beta = 0.07 (first-person perspective), beta = 0.09 (third-person perspective) | Dechant et al. (2021a) |
|  | Target error (i.e., distance to expected target when task finished) | Sig. positive relation with social anxiety for customized avatar (beta = 0.14* for first- and third-person perspective), sig. negative relation for predefined avatar (beta = -0.42* (first-person perspective), beta = -0.27* (third-person perspective)) | Dechant et al. (2021a) |
| **Path length** | Path length (i.e., absolute travelled distance per trial) | Sig. positive relation with social anxiety for predefined avatars (beta = .27* for first-person and beta = 0.18* for third-person perspective), (sig.) negative relation with social anxiety for customized avatars (beta = - 0.15* for first-person and beta = -0.10 for third-person perspective) | Dechant et al. (2021a) |
|  | Path length (i.e., absolute travelled distance per trial) - implicit | Non sig. predictor of social anxiety (beta = 0.09) | Dechant et al. (2021b) |
| **Time in room** | Time in room from start to completion of a trial | Sig. negative predictor of social anxiety for custumized avatar in first-person perspective (beta = -0.15*), but no sig. relations for other conditions (beta = 0.03 for custumized avatar in third-person perspective; personalized avatar: beta = -0.06 (first-person perspective), beta = -0.02 (third-person perspective)) | Dechant et al. (2021a) |
|  |  | Sig. positive predictor of trait social anxiety (beta = 0.23*) | Dechant et al. (2021b) |

*Notes.* Where significance of the results is not reported (by sig. or non sig. description), the information was not available in the reviewed paper. NPC = non-player character.
